# ESBL-producing *Escherichia coli* in wastewater from German slaughterhouses

**DOI:** 10.1016/j.onehlt.2025.101189

**Published:** 2025-09-03

**Authors:** Jette F. Kleist, Lisa Bachmann, Christiane Huxdorff, Elias Eger, Katharina Schaufler, Judith Wedemeyer, Timo Homeier-Bachmann

**Affiliations:** a University of Applied Science Neubrandenburg, Germany; b Research Institute of Farm Animal Biology (FBN), Institute of Nutritional Physiology “Oskar Kellner”, Germany; c Greenpeace e.V., 20547 Hamburg, Germany; d Helmholtz Centre for Infection Research, Helmholtz Institute for One Health, Department Epidemiology and Ecology of Antimicrobial Resistance, Greifswald, Germany; e University Medicine Greifswald, Greifswald, Germany; f Friedrich-Loeffler-Institute, Institute of Epidemiology, Greifswald-Insel Riems, Germany

**Keywords:** ESBL-*E. coli*, Slaughterhouse, Wastewater, Resistance, Whole genome sequencing, Clonal analysis

## Abstract

The increasing prevalence of ESBL-producing *Escherichia (E.) coli* (ESBL-*E. coli*) poses a significant threat to human and animal health, with livestock identified as a critical reservoir. This study examined the occurrence and diversity of ESBL-*E. coli* in wastewater from four German slaughterhouses (pig and poultry) over two sampling periods. The primary objective of the study was to determine whether these facilities have a persistent colonization of AMR bacteria or whether their presence fluctuates daily depending on which animals are processed. Using selective media and subsequent whole-genome sequencing, 47 wastewater samples were analyzed and 86 ESBL-*E. coli* strains were isolated, 40 of which were multidrug-resistant (MDR). Specifically, 27 out of 37 isolates from poultry and 13 out of 49 isolates from pig slaughterhouses were confirmed as MDR. In contrast to the expectation of a long-term persistent colonization of the abattoirs, our results indicate a daily introduction of ESBL-*E. coli* by the animals processed. The consistent detection of these strains in wastewater from slaughterhouses underlines the role of these facilities as important hotspots for the dissemination of AMR bacteria in the environment. This underscores the urgent need for enhanced surveillance and control measures to curtail the spread of antimicrobial resistance.

## Highlights

• 86 ESBL-*E. coli* strains were obtained from 47 slaughterhouses wastewater samples.
• 46,5 % of the ESBL-*E. coli* strains were multidrug resistant (MDR).
• MDR-ratio in poultry (72.97 %) was higher than in pig slaughterhouses (26.53 %).
• No hints for permanent colonization of slaughterhouses were found.

## 1 Introduction

Antimicrobials are among the most important tools in the treatment of bacterial infections. The continuous development of antimicrobial-resistant (AMR) bacteria and their rapid spread in the environment poses a serious threat for global health. In consequence, the WHO has established a global priority list of important AMR-bacteria, including extended-spectrum β-lactamase (ESBL)-producing Enterobacterales, because of the global importance of this resistant phenotype as a major cause of life-threatening infections [[1], [2], [3], [4]]. ESBL-producing bacteria excrete enzymes that are able to hydrolyze β-lactam-antibiotics, including those of newer generations such as the broad-spectrum cephalosporins of the 3rd- and 4th-generation [5].

One of the most prevalent ESBL-producing Enterobacterales is *Escherichia (E.) coli*, which is capable of producing highly efficient β-lactamases [6]. In the most recent issue of the WHO's Bacterial Priority Pathogens List (BPPL), third-generation cephalosporin-resistant *E. coli* has ascended to second place, superseded by carbapenem-resistant *Klebsiella pneumoniae* [4].

The widespread use of antimicrobials in livestock farming is common practice and is intended to improve animal health, welfare and productivity [7]. The most commonly used antimicrobial classes are tetracyclines, penicillins, macrolides and polymyxins in German pig farming and aminoglycosides, lincosamides, polypeptides and β-lactams in poultry farming [8,9]. The total amount of antibiotics used in German livestock farming in 2021 was approximately 601 t [10]. For 2023, the total amount has fallen to 529 t [11]. Since any contact between bacteria and antimicrobials potentially promotes the emergence of AMR, it is not surprising that livestock farming has become a reservoir of AMR-bacterial strains [12,13]. However, the spread of ESBL-producers is not the only risk posed by livestock farming in this context. In addition, occurrence of multidrug-resistant (MDR) bacteria, i.e. Gram-negative bacteria that exhibit resistance towards at least three antimicrobial classes [14] in the context of pig and poultry farming, increases this threat [15,16].

These AMR bacteria, which originate from pigs or poultry, can be spread via wastewater discharged from slaughterhouses [15,16]. In 2022, about 47 million pigs and 631 million poultry were slaughtered in Germany [17,18]. During the slaughtering process, large amounts of wastewater are produced, which could potentially be contaminated with AMR-bacteria [19]. The in-house wastewater treatment plants (WWTP) of the slaughterhouses remove organic contamination from the wastewater as required by the Directive 2010/75/EU and the Decision (EU) 2016/902 of the European Union [20]. However, this treatment does not target bacteria. Thereafter, the wastewater containing these bacteria is discharged into public water bodies, which can lead to the uncontrolled spread of AMR bacteria in the environment [21,22].

The objective of this study was to assess the discharge of AMR-bacteria from slaughterhouse abattoirs and the associated risk to the environment. To this end the occurrence and diversity of antimicrobial-resistant *E. coli* in wastewater from four German slaughterhouses was examined over two sets of three consecutive days. The objective was to ascertain whether certain AMR-bacteria exhibit a high degree of recurrence in the samples, indicating a persistent colonization of the abattoirs and, consequently, a persistent discharge of these bacteria, or whether the occurrence of these bacteria varies on a daily basis depending on the animals being processed. It is imperative to acknowledge the possibility of continuous environmental discharge of AMR-bacteria resulting from persistent colonization within abattoirs. This is of particular concern given the evidence from numerous studies that have already demonstrated the capacity of WWTPs to function as continuous sources of resistant bacteria [15,[23], [24], [25], [26]]. A permanent discharge of AMR-bacteria into the environment has been demonstrated to have the potential to increase the risk of the development and spread of new resistance genes as well as the emergence of new pathogens [27,28].

## 2 Material and methods

Sampling Locations and Sample Collection.

Samples were collected by collection teams from Greenpeace e.V. in January and February 2022. Samples were obtained on two sets of three consecutive days. The initial sampling occurred on three consecutive days, followed by a repetition of the same procedure two weeks later, on another three consecutive days. Samples were taken from four slaughterhouses, two specialized on poultry processing (slaughterhouse A and B) and two on pigs (slaughterhouse C and D). Each sample was taken in duplicate. On day one, only one sample was taken at slaughterhouse D, so no duplicate was taken. The four slaughterhouses that were sampled had previously been the subject of a study investigating antibiotic-resistant Enterobacterales in wastewater [15].

Samples were collected directly from the WWTP outlet of each slaughterhouse, just before the effluent was released into the receiving water body/river. Each sample contained approximately 500 mL and was collected in a sterile plastic container (Carl Roth GmbH, Karlsruhe, Germany). The containers were kept refrigerated during transport and delivered to the Friedrich-Loeffler-Institut (FLI) on the day of sampling.

### 2.1 Isolation of bacteria and identification

The water samples were subjected to a preliminary filtration process using sterilized gauze (PZN: 04046708, FESMED Verbandmittel GmbH, Frankenberg/Sa., Germany) with the objective of removing fine particulate matter. The pre-filtered water was devoid of sediment, larger particles and the majority of smaller particles. Subsequently, each sample was filtered using the EZ-Fit filtration system equipped with 0.45 μm pore-sized filter membranes (merckmillipore, Darmstadt, Germany). Post-filtration, these membranes were transferred to 10 mL lysogeny broth (LB) (Carl Roth GmbH, Karlsruhe, Germany) supplemented with 2 μg/mL cefotaxime (VWR International, Darmstadt, Germany) and incubated overnight at 37 °C while shaking at 200 rpm. For each sample, 100 μL of the suspension were plated on chromogenic agar plates CHROMagar™ ESBL, CHROMagar™ COL-APSE and CHROMagar™ mSuperCARBA (MAST Diagnostica GmbH, Reinfeld, Germany) and incubated overnight at 37 °C. To examine whether certain *E. coli* strains were dominant in the samples tested and to gain insight into the bacterial diversity, four potential antibiotic resistant colonies of *E. coli* (red-purple colonies) each per sample were sub-cultivated until pure cultures were obtained. Colonies selected for sub-cultivation were chosen randomly, without considering specific morphological characteristics. Pure colonies were used for further verification and characterization.

### 2.2 Antimicrobial susceptibility testing (AST)

The bacterial strains were subjected to antibiotic susceptibility testing (AST) using the VITEK2 system (bioMérieux, Nürtingen, Germany) with software version 9.02 and the AST-N389-card, in accordance with the manufacturer's guidelines. This AST-card contained an ESBL confirmation test. First-, third-, fourth- and fifth generation cephalosporins cefalexin, cefotaxime, ceftazidime ceftolozan and cefepime were used alone or combined with tazobactam. A decrease in growth when combined with tazobactam indicated potential ESBL production. The AST results of the VITEK2 system were based on the European Committee on Antimicrobial Susceptibility Testing (EUCAST) breakpoint tables for interpretation of MICs and zone diameters (Version 11.0, 2021. https://www.eucast.org/). To ensure the integrity of our AST results, the antibiotic-susceptible strains *E. coli* ATCC25922 and *Pseudomonas aeruginosa* ATCC27853, specified by the manufacturer, were used for quality control.

### 2.3 Sequence analysis

DNA extraction was performed using the MasterPure™ DNA Purification Kit for Blood, Version II (Lucigen, Middleton, USA). DNA from all bacterial strains was shipped to the Microbial Genome Sequencing Center (MiGS) in Pittsburgh, PA, USA, where whole-genome-sequencing (WGS) was carried out as described elsewhere [15]. In brief, the de novo genome assemblies of the sequencing data were conducted by employing the assembly pipeline shovill (https://github.com/tseemann/shovill) in combination with SPAdes [29]. The in-silico multi-locus sequence typing (MLST) and antibiotic resistance/virulence gene detection were carried out using mlst v. 2.19.0 (https://github.com/tseemann/mlst) and ABRicate v. 1.0.0 (https://github.com/tseemann/abricate), respectively. Both tools rely on 3rd party public databases (e.g., PubMLST [30], ResFinder [31], ARGANNOT [32]).

### 2.4 Clonal expansion

Clonal expansion analysis was performed for 12 ESBL-producing *E. coli* ST410 isolates and 16 ESBL-producing *E. coli* ST4981 isolates, including two isolates originating from slaughterhouses A and B in 2020 [15], to construct their core single nucleotide polymorphisms (SNP) phylogeny. To create an alignment of the WGS-sequences, the reads were mapped against a reference genome (3080/ST410 and 2916/ST4981) using Snippy v.4.6.0 (https://github.com/tseemann/snippy). The alignment was then processed using Gubbins v.2.4.1 [33] and snp-sites v.2.5.1 [34]. FastTree v.2.1.11 (http://www.microbesonline.org/fasttree/) [35] was used to infer maximum-likelihood phylogenetic trees from the alignments. Midpoint-rooting of the maximum-likelihood trees was performed in iTOL v.7.0 after [36]. To convert the FASTA alignment to a SNP distance matrix, Snp-dists v.0.8.2 (https://github.com/tseemann/snp-dists) was used. The distance matrix is depicted as a heat map diagram.

### 2.5 *Virulence testing in Galleria mellonella* larvae model

The virulence potential of six representative ST4981 isolates (i.e., 2916, 2944, 2980, 2987, 2993 and 3085), selected on the basis of phylogenetic analysis, was evaluated using the *Galleria mellonella* larvae model. To provide a broader context, an additional ST4981 isolate (469), originating from slaughterhouse A in 2020 [15], was included. In brief, overnight cultures of each isolate were diluted 1:100 in LB and grown until turbidity reached OD_600_ = 0.1. Thereafter, bacterial suspensions were washed twice with PBS. Randomized groups of 10 larvae (Tierbedarf Baig, Neumünster, Germany) were infected according to previously published protocol [37]. To account for possible injection trauma, 10 larvae were injected with 10 μL sterile PBS for each experiment, while 10 larvae were used as non-injected controls. Larvae were incubated at 37 °C for 72 h with mortality monitoring at 12 h, 24 h, 48 h and 72 h post-infection. For reference, a laboratory strain of *E. coli* K12 (negative control) and an *E. coli* isolate from ST58 (PBIO3502) derived from meat (positive control) [38] were used and a Kaplan-Meier plot of survival rates was generated.

## 3 Results

### 3.1 Bacteriological examination

On all six sampling days, bacterial growth, which was morphologically indicative of *E. coli*, was obtained on CHROMagar™ ESBL plates from wastewater outlets of the abattoirs from three out of four slaughterhouses. In samples from slaughterhouse A, a potential growth of *E. coli* was present only in two of six samplings. For the CHROMagar™ COL-APSE-plates, bacterial growth, suspicious for *E. coli*, was only detected in samples from two slaughterhouses on five (slaughterhouse B) and two (slaughterhouse D) days, respectively. No bacterial growth was detected on the CHROMagar™ mSuperCARBA media (Table 1). Based on the color and morphology of the colonies, four suspected colonies each were sub-cultivated in order to represent the diversity of the bacteria. In total, 86 *E. coli* isolates were obtained (slaughterhouse A: *n* = 8; slaughterhouse B: *n* = 29; slaughterhouse C: *n* = 24; slaughterhouse D: *n* = 25) and underwent further analyses. Species identification was confirmed by WGS (Supplementary Table 2).

**Table 1:** Growth on selective media.

		Selective Media		
Slaughterhouse	Sampling Date	ESBL	COL-APSE	mSuperCARBA
A (poultry)	24.01.2022	**−**	**−**	**−**
	25.01.2022	**+**	**−**	**−**
	26.01.2022	**+**	**−**	**−**
	07.02.2022	**−**	**−**	**−**
	08.02.2022	**−**	**−**	**−**
	09.02.2022	**−**	**−**	**−**
B (poultry)	24.01.2022	**+**	**+**	**−**
	25.01.2022	**+**	**−**	**−**
	26.01.2022	**+**	**+**	**−**
	07.02.2022	**+**	**+**	**−**
	08.02.2022	**+**	**+**	**−**
	09.02.2022	**+**	**+**	**−**
C (swine)	24.01.2022	**+**	**−**	**−**
	25.01.2022	**+**	**−**	**−**
	26.01.2022	**+**	**−**	**−**
	07.02.2022	**+**	**−**	**−**
	08.02.2022	**+**	**−**	**−**
	09.02.2022	**+**	**−**	**−**
D (swine)	24.01.2022	**+**	**−**	**−**
	25.01.2022	**+**	**−**	**−**
	26.01.2022	**+**	**+**	**−**
	07.02.2022	**+**	**−**	**−**
	08.02.2022	**+**	**−**	**−**
	09.02.2022	**+**	**+**	**−**

### 3.2 Antimicrobial susceptibility testing (AST)

All ESBL-suspicious isolates [n = 86] showed ESBL-phenotypes, as they were resistant to the first-, third- and fourth-generation cephalosporins tested (cefalexin, cefepime, cefotaxime, ceftazidime), as well as to penicillins (ampicillin) and aminopenicillins (amoxicillin). In addition, all isolates [*n* = 86] showed resistance towards the combination of amoxicillin and clavulanic acid. Against the combination of piperacillin and tazobactam, four isolates (4.65 %) were resistant, one isolate (1.16 %) was susceptible, while the remaining isolates (94.19 % [*n* = 81]) were classified as susceptible to increased exposure. For the last-resort antibiotic, colistin, 16.28 % [*n* = 14] of the isolates were phenotypically resistant. To the fifth-generation cephalosporin, ceftolozane combined with tazobactam, all isolates [n = 86] were susceptible.

Out of the 86 isolates examined, 46.51 % [*n* = 40] corresponded to the definition of MDR. For the bacterial strains isolated from poultry abattoirs, 27 out of 37 isolates (72.97 %) were MDR. Out of the pig abattoirs, 13 of the 49 isolates (26.53 %) were identified as MDR. Details about the AST results are given in Supplementary Table 1.

### 3.3 Whole-Genome sequencing (WGS)

The results of multilocus sequence typing and prediction of AMR determinants using (including tool/database) based on genomic analysis are summarized in Supplementary Table 2. The WGS analysis revealed 30 different sequence types (ST) for the *E. coli* isolates.

In all 86 isolates, ESBL-genes were detected. The most frequently occurring CTX-M-genes were *bla*_CTX-M-15_ (41.86 % [*n* = 36]) and *bla*_CTX-M-1_ (38.37 % [*n* = 33]). Furthermore, the ESBL-genes *bla*_CTX-M-55_ (8/86), *bla*_CTX-M-27_ (1/86), *bla*_TEM-1B_ (30/86), *bla*_TEM-106_ (11/86), *bla*_TEM-1C_ (4/86), *bla*_TEM-141_ (2/86), *bla*_SHV-12_ (5/86), *bla*_CMY-2_ (2/86), *bla*_OXA-1_ (2/86) and *bla*_CARB-16_ (2/86) were detected. All 86 isolates harbored the resistance gene *mdf*, belonging to a transport protein, which conveys resistance to a broad spectrum of antibiotics and toxic substances [39]. In 14 isolates (16.28 %), the gene *mcr-1* was detected, which provides resistance to the last-resort antibiotic colistin. Resistance genes associated with resistances against aminoglycosides, quinolones, phenicol, tetracyclines, fosfomycin, sulfonamides, macrolides and diaminopyrimidines were also detected as shown in Supplementary Table 2.

To detect persistent bacteria, isolates occurring on consecutive days and belonging to the same ST were selected. Thirty-one *E. coli* isolates with same STs appearing on two/three consecutive days were found, suggesting that they could be persistent bacteria. These isolates belonged to 7 different STs (ST10, ST58, ST75, ST88, ST410, ST453, ST4981). Phenotypic and genotypic resistance data were compared to determine whether these were clones. The comparison revealed similar phenotypic and genotypic resistance patterns for 12 isolates belonging to ST410 and 14 isolates belonging to ST4981, which occurred on consecutive days. Subsequently, a phylogeny analysis was conducted for the isolates in question.

### 3.4 Clonal expansion

**Fig. 1:**
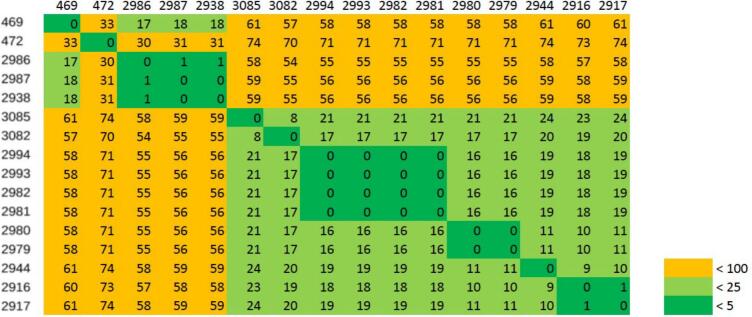
Core SNP matrix of ST4981 isolates.

Phylogeny analysis of ST410 and ST4981 isolates revealed distinct clustering patterns with varying levels of genetic similarity. The core genome of the analyzed ST4981 isolates (Fig. 1) was 4,534,575 bp, and two major phylogenetic clusters were identified. The first cluster included the isolates 469 (slaughterhouse A) and 472 (slaughterhouse B) from the previous study of Homeier-Bachmann et al. [15], as well as the isolates 2938, 2986, and 2987 (slaughterhouse C, sampling days 2 and 3), with 0–33 SNPs. The second cluster contained the isolates 2916, 2917 (sampling day 1), 2944 (sampling day 2), 2979, 2980, 3082 and 3085 (sampling day 3) from slaughterhouse B, and 2981, 2982, 2993 and 2994 (slaughterhouse A, sampling day 3), with genetic variations of 0–24 SNPs. Within the second cluster, the isolates 2981, 2982, 2993 and 2994 were identical (0 SNPs difference). as well as the isolates 2979 and 2980. The genetic variation between isolates 2916 and 2917 was 1 SNP.

**Fig. 2:**
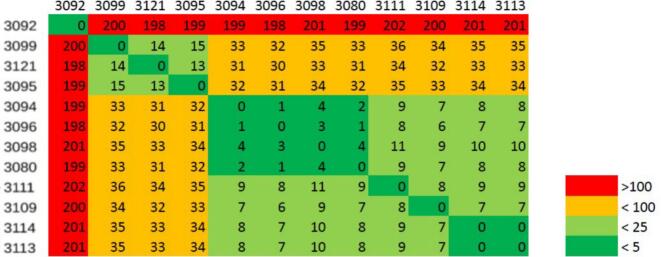
Core SNP matrix of ST410 isolates.

For ST410, a 4,571,635 bp core genome and two major phylogenetic clusters were found (Fig. 2). The first cluster was formed by isolates 3095, 3099 (slaughterhouse B, sampling day 5) and 3121 (slaughterhouse D, sampling day 6), with genetic variations between 0 and 15 SNPs. The second cluster included isolates 3080 (sampling day 4), 3094, 3096, 3098 (sampling day 5), 3109, 3111, 3113 and 3114 (sampling day 6) from slaughterhouse B with genetic variations between 0 and 11 SNPs. Isolates 3080, 3094, 3096 and 3098 only differed by 0–4 SNPs, the isolates 3113 and 3114 were identical.

### 3.5 Virulence testing

**Fig. 3:**
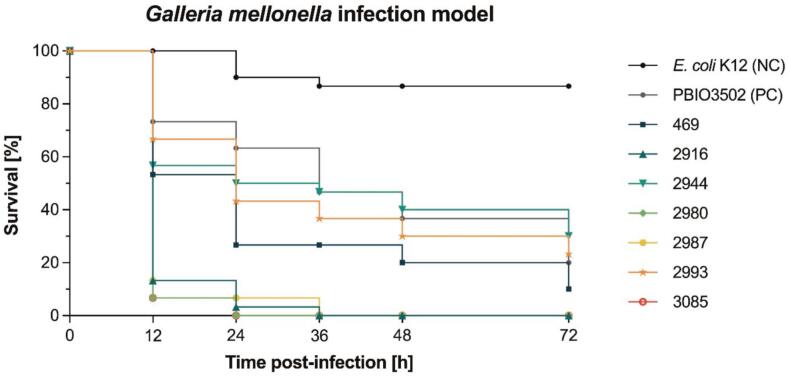
Kaplan-Meier plot of larval survival in *Galleria mellonella* infection model (*n* = 30 larvae). The results are expressed as mean percentage survival after injection of 10^5^ CFU per larva. NC, negative control, PC, positive control.

To investigate the pathogenic potential of six representative ST4981 isolates, an in vivo infection assay using the *G. mellonella* larvae model was performed (Fig. 3). This analysis revealed two distinct clusters of isolates, each with distinctly different virulence characteristics. The first cluster, consisting of the isolates 2916, 2980, 2987, and 3085, exhibited a high level of virulence, as evidenced by a rapid decrease in larval survival. Within 12 h post-infection, survival rates dropped to between 6.7 and 13.3 %. Progression of the infection resulted in complete mortality of the larvae between 24 and 36 h.

In contrast, the second cluster, which included isolates 469, 2944, and 2993, exhibited significantly lower pathogenic potential, comparable to the reference strain PBIO3502. After 12 h post-infection, larval survival in this group was reduced to between 53.3 % and 66.7 %. Over the 72-h observation period, survival rates gradually decreased, eventually reaching a range of 10 % to 30 %.

## 4 Discussion

Wastewater from four German slaughterhouses was analyzed over two sampling periods, each comprising three consecutive days, to examine the occurrence and diversity of ESBL-*E. coli* in wastewater, assessing whether these abattoirs exhibit persistent colonization or whether the presence of AMR bacteria fluctuates depending on the animals processed. The results clearly demonstrate that ESBL-*E. coli* are prevalent in both pig and poultry slaughterhouse wastewater, since 86 isolates were found and identified. This finding aligns with previous research, which has documented the presence of these bacteria in both livestock farming and WWTP [7,15,16,21,22,40].

High levels of ESBL-*E. coli* (*n* = 86) were detected in the majority of the sampled wastewater. With the exception of slaughterhouse A, where ESBL-*E. coli* were found only on two out of six sampling days, all other WWTP consistently emitted resistant bacteria into the environment on all sampling days. This indicates regular AMR contamination of the wastewater and is in line with other research, which also reports high prevalences in slaughterhouse abattoir effluent [15,16,21,22,40]. While Homeier-Bachmann et al. [15] reported higher prevalence of AMR bacteria in poultry abattoirs, our findings point towards a lower prevalence of ESBL-*E. coli* in wastewater from poultry compared to pig slaughterhouses. This discrepancy appears to be largely driven by slaughterhouse A, which had particularly low AMR levels. Due to a lack of information about slaughterhouse A, such as its hygiene protocols or wastewater treatment processes, possible reasons for this finding are unclear.

Among the 86 isolates found, 46.51 % showed an MDR-phenotype, i.e., were resistant to three or more antimicrobial classes. The occurrence of MDR-bacteria was higher in poultry (72.97 %) than in pig (26.53 %) abattoirs. 16.28 % (*n* = 14) of the isolates found had phenotypic resistance against the antibiotic colistin, which is classified as a human last-resort antibiotic. Still, its use is common in veterinary medicine, especially in pig and cattle production [[41], [42], [43]]. In poultry farming, colistin is used less frequently, but mainly in the treatment of colibacillosis [44]. Despite this, 13 of the 14 colistin-resistant isolates originated from poultry slaughterhouses. Similar findings have been reported by Homeier-Bachmann et al. and Savin et al. [15,40], where the percentage of colistin-resistant isolates was higher in samples from poultry slaughterhouses compared to pig slaughterhouses. Since nothing is known about the slaughtered animals, such as their origin, antibiotic usage history or farm-level practices, no statements can be made about possible reasons for this finding.

WGS identified 30 different STs among the isolates, some of which are known as pathogenic strains, including some recognized as global high-risk clones associated with extraintestinal pathogenic *E. coli* (ExPEC) (ST1011, ST410, ST10, ST117, ST58, ST131) [[45], [46], [47], [48], [49], [50]]. These strains pose a potential health threat if released into public water bodies.

Isolates of STs 410 and 4981 underwent phylogenetic analysis. ST410-isolates 3095, 3099 (slaughterhouse B), and 3121 (slaughterhouse D) were found to be highly genetically similar, with genetic variations between 13 and 15 SNPs, despite originating from different livestock species. As the number of core genome SNPs was below 17, the isolates meet the clone definition established by Ludden et al. [51]. This suggests that ST410 is highly adaptable to different livestock environments. Given that ST410 is a well-known, high-risk clone associated with ExPEC, with known zoonotic potential due to its high adaptability in both humans and animals [52,53]. This distinct adaptability could explain why genetically similar strains were found in pig as well as poultry slaughterhouses. ST4981-isolates 2916, 2917, 2944, 2979, 2980, 3082, 3085 (slaughterhouse B), and 2981, 2982, 2993, and 2994 (slaughterhouse A) showed high genetic similarities despite originating from different slaughterhouses. Little is reported about ST4981, but it is mentioned in the context of pig farming, human healthcare settings and wastewater systems [15,[54], [55], [56]]. To assess its potential pathogenic behavior, virulence testing using *Galleria mellonella* was performed and showed pathogenic behavior for all isolates tested, with medium to high mortality (Fig. 3). Due to its diverse occurrence in both pig and poultry abattoirs, and its pathogenic potential, ST4981 can be assumed to be a successful clone in livestock farming, which also poses a potential health risk for animals and humans.

Our findings do not provide clear evidence of persistent colonization in the abattoirs tested. While genetically similar isolates were detected, they were only identified over a maximum period of three consecutive days, which suggests that the bacteria are not able to persist long term. This is why it can be assumed that the prevalence of ESBL-*E. coli* changes daily and is dependent on the animals slaughtered. While no clear evidence of persistent colonization was found, the consistent detection of ESBL-*E. coli* in slaughterhouse wastewater highlights an ongoing health concern. The present study is subject to certain limitations arising from the restriction of the research to four slaughterhouses and the implementation of only two short sampling periods. Consequently, it is not possible for the present study to provide comprehensive conclusions on the long-term persistence of AMR or its overall shedding due to slaughterhouses. In order to achieve this objective, it is essential to undertake follow-up studies, preferably in the form of longitudinal studies. The uncontrolled spread of AMR bacteria into the environment poses a major potential risk in terms of the One Health-approach. Once discharged into public water bodies, these bacteria can persist in the environment, facilitating the exchange of resistance genes between the environment, animals, and humans, contributing to the spread of AMR and threatening public health.

## 5 Conclusion

This study demonstrates the consistent presence of ESBL-producing *E. coli* in wastewater from pig and poultry slaughterhouses, indicating the regular release of AMR bacteria into the environment. Although genetically similar strains were identified, there was no clear evidence of long-term persistent colonization within the abattoirs, which suggests that prevalence fluctuates according to the number of animals processed. Despite the limited number of slaughterhouses and sampling periods, these findings emphasise the ongoing risk of AMR dissemination from slaughterhouse wastewater, underlining the need for continued monitoring and mitigation measures within the One Health framework.

## CRediT authorship contribution statement

**Jette F. Kleist:** Writing – original draft, Visualization, Validation, Formal analysis, Data curation. **Lisa Bachmann:** Writing – original draft, Visualization, Validation, Formal analysis. **Christiane Huxdorff:** Writing – review & editing, Investigation. **Elias Eger:** Writing – review & editing, Visualization, Investigation. **Katharina Schaufler:** Writing – review & editing, Validation, Supervision. **Judith Wedemeyer:** Writing – review & editing, Visualization, Formal analysis. **Timo Homeier-Bachmann:** Writing – review & editing, Validation, Supervision, Project administration, Investigation, Data curation.

## Funding

This research did not receive any specific grant from funding agencies in the public, commercial, or not-for-profit sectors.

## Declaration of competing interest

We confirm that this manuscript is original, has not been published elsewhere, and is not under consideration for publication elsewhere. All authors have approved the submission and have no conflicts of interest to disclose.

## Data Availability

Data for this study were deposited in the European Nucleotide Archive (ENA) at EMBL-EBI under accession number PRJEB87378

## Appendix A Supplementary data

Supplementary material 1 Phenotypic resistance profiles of isolates. R = resistant, S = sensitive Image 1

Supplementary material 2 Resuslts of the WGS analysis Image 2
