# Supplementary material for: ESBL-producing Escherichia coli in wastewater from German slaughterhouses
Source: One Health. 2025 Sep 3;21:101189. doi: 10.1016/j.onehlt.2025.101189 (PMC12451283; doi:10.1016/j.onehlt.2025.101189)
Supplement: Supplementary file 1 — Supplementary material 1 Phenotypic resistance profiles of isolates. R = resistant, S = sensitive [file mmc1.docx]

Supplementary table 1. Phenotypic resistance profiles of isolates. R = resistant, S = sensitive

| Designation | Slaughterhouse | Day of sampling | Medium | ESBL | MDR | Amoxicillin | Ampicillin | Amoxicillin/clavulanic acid | Piperacillin/tazobactam | Cefalexine | Cefotaxime | Ceftazidime | Ceftolozane/tazobactam | Cefepim | Aztreonam | Imipenem | Meropenem | Amikacin | Gentamicin | Tobramycin | Ciprofloxacin | Tigecyclin | Fosfomycine | Colistin | Trimethoprim/sulfamethoxazole |
| --- | --- | --- | --- | --- | --- | --- | --- | --- | --- | --- | --- | --- | --- | --- | --- | --- | --- | --- | --- | --- | --- | --- | --- | --- | --- |
| 2946 | A (poultry) | 2 | ESBL | + | - | R | R | R | S | R | R | R | S | R | R | S | S | S | S | S | S | S | S | S | S |
| 2947 | A (poultry) | 2 | ESBL | + | - | R | R | R | S | R | R | R | S | R | R | S | S | S | S | S | S | S | S | S | S |
| 2948 | A (poultry) | 2 | ESBL | + | - | R | R | R | S | R | R | R | S | R | R | S | S | S | S | S | S | S | S | S | S |
| 2949 | A (poultry) | 2 | ESBL | + | - | R | R | R | S | R | R | R | S | R | R | S | S | S | S | S | S | S | S | S | S |
| 2981 | A (poultry) | 3 | ESBL | + | + | R | R | R | S | R | R | R | S | R | S | S | S | S | S | S | R | S | S | S | R |
| 2982 | A (poultry) | 3 | ESBL | + | + | R | R | R | S | R | R | R | S | R | S | S | S | S | S | S | R | S | S | S | R |
| 2993 | A (poultry) | 3 | ESBL | + | + | R | R | R | S | R | R | R | S | R | S | S | S | S | S | S | R | S | S | S | R |
| 2994 | A (poultry) | 3 | ESBL | + | + | R | R | R | S | R | R | R | S | R | S | S | S | S | S | S | R | S | S | S | R |
| 2916 | B (poultry) | 1 | ESBL | + | + | R | R | R | S | R | R | R | S | R | S | S | S | S | S | S | R | S | S | S | R |
| 2917 | B (poultry) | 1 | ESBL | + | + | R | R | R | S | R | R | R | S | R | S | S | S | S | S | S | R | S | S | S | R |
| 2942 | B (poultry) | 2 | ESBL | + | - | R | R | R | S | R | R | R | S | R | R | S | S | S | S | S | S | S | S | S | S |
| 2943 | B (poultry) | 2 | ESBL | + | - | R | R | R | S | R | R | R | S | R | R | S | S | S | S | S | S | S | S | S | S |
| 2944 | B (poultry) | 2 | ESBL | + | + | R | R | R | S | R | R | R | S | R | S | S | S | S | S | S | R | S | S | S | R |
| 2945 | B (poultry) | 2 | ESBL | + | - | R | R | R | S | R | R | R | S | R | R | S | S | S | S | S | S | S | S | S | S |
| 2979 | B (poultry) | 3 | ESBL | + | + | R | R | R | S | R | R | R | S | R | S | S | S | S | S | S | R | S | S | S | R |
| 2980 | B (poultry) | 3 | ESBL | + | + | R | R | R | S | R | R | R | S | R | S | S | S | S | S | S | R | S | S | S | R |
| 2983 | B (poultry) | 3 | ESBL | + | - | R | R | R | S | R | R | R | S | R | R | S | S | S | S | S | S | S | S | S | S |
| 2984 | B (poultry) | 3 | ESBL | + | - | R | R | R | S | R | R | R | S | R | R | S | S | S | S | S | S | S | S | S | S |
| 3033 | B (poultry) | 3 | COL | + | + | R | R | R | S | R | R | R | S | R | R | S | S | S | R | R | R | S | S | R | R |
| 3080 | B (poultry) | 4 | ESBL | + | + | R | R | R | S | R | R | R | S | R | R | S | S | S | S | S | R | S | S | R | R |
| 3081 | B (poultry) | 4 | ESBL | + | + | R | R | R | S | R | R | R | S | R | S | S | S | S | S | S | R | S | S | S | R |
| 3082 | B (poultry) | 4 | COL | + | + | R | R | R | S | R | R | R | S | R | R | S | S | S | S | S | R | S | S | R | R |
| 3083 | B (poultry) | 4 | ESBL | + | + | R | R | R | S | R | R | R | S | R | R | S | S | S | S | S | R | S | R | S | R |
| 3084 | B (poultry) | 4 | ESBL | + | + | R | R | R | S | R | R | R | S | R | R | S | S | S | S | S | R | S | R | S | R |
| 3085 | B (poultry) | 4 | COL | + | + | R | R | R | S | R | R | R | S | R | R | S | S | S | S | S | R | S | S | R | S |
| 3094 | B (poultry) | 5 | COL | + | + | R | R | R | S | R | R | R | S | R | R | S | S | S | S | S | R | S | S | R | R |
| 3095 | B (poultry) | 5 | COL | + | + | R | R | R | S | R | R | R | S | R | R | S | S | S | S | S | R | S | S | R | R |
| 3096 | B (poultry) | 5 | COL | + | + | R | R | R | S | R | R | R | S | R | R | S | S | S | S | S | R | S | S | R | R |
| 3097 | B (poultry) | 5 | ESBL | + | - | R | R | R | S | R | R | R | S | R | S | S | S | S | S | S | S | S | S | S | S |
| 3098 | B (poultry) | 5 | COL | + | + | R | R | R | S | R | R | R | S | R | R | S | S | S | S | S | R | S | S | R | R |
| 3099 | B (poultry) | 5 | COL | + | + | R | R | R | S | R | R | R | S | R | R | S | S | S | S | S | R | S | S | R | R |
| 3109 | B (poultry) | 6 | COL | + | + | R | R | R | S | R | R | R | S | R | R | S | S | S | S | S | R | S | S | R | R |
| 3110 | B (poultry) | 6 | ESBL | + | + | R | R | R | S | R | R | R | S | R | S | S | S | S | S | S | R | S | S | S | R |
| 3111 | B (poultry) | 6 | COL | + | + | R | R | R | S | R | R | R | S | R | S | S | S | S | S | S | R | S | S | R | R |
| 3112 | B (poultry) | 6 | ESBL | + | + | R | R | R | S | R | R | R | S | R | S | S | S | S | S | S | R | S | S | S | R |
| 3113 | B (poultry) | 6 | COL | + | + | R | R | R | S | R | R | R | S | R | R | S | S | S | S | S | R | S | S | R | R |
| 3114 | B (poultry) | 6 | COL | + | + | R | R | R | S | R | R | R | S | R | R | S | S | S | S | S | R | S | S | R | R |
| 2914 | C (pSg) | 1 | ESBL | + | - | R | R | R | S | R | R | R | S | R | S | S | S | S | S | S | S | S | S | S | R |
| 2915 | C (pSg) | 1 | ESBL | + | - | R | R | R | S | R | R | R | S | R | R | S | S | S | S | S | S | S | S | S | S |
| 2920 | C (pSg) | 1 | ESBL | + | + | R | R | R | S | R | R | R | S | R | S | S | S | S | S | S | R | S | S | S | R |
| 2921 | C (pSg) | 1 | ESBL | + | + | R | R | R | S | R | R | R | S | R | S | S | S | S | S | S | R | S | S | S | S |
| 2938 | C (pSg) | 2 | ESBL | + | + | R | R | R | S | R | R | R | S | R | R | S | S | S | S | S | R | S | S | S | S |
| 2939 | C (pSg) | 2 | ESBL | + | - | R | R | R | S | R | R | R | S | R | R | S | S | S | S | S | S | S | S | S | R |
| 2940 | C (pSg) | 2 | ESBL | + | - | R | R | R | S | R | R | R | S | R | S | S | S | S | S | S | S | S | S | S | R |
| 2941 | C (pSg) | 2 | ESBL | + | + | R | R | R | S | R | R | R | S | R | S | S | S | S | S | S | R | S | S | S | S |
| 2985 | C (pSg) | 3 | ESBL | + | - | R | R | R | S | R | R | R | S | R | S | S | S | S | S | S | S | S | S | S | S |
| 2986 | C (pSg) | 3 | ESBL | + | + | R | R | R | S | R | R | R | S | R | R | S | S | S | S | S | R | S | S | S | S |
| 2987 | C (pSg) | 3 | ESBL | + | + | R | R | R | S | R | R | R | S | R | R | S | S | S | S | S | R | S | S | S | S |
| 2988 | C (pSg) | 3 | ESBL | + | - | R | R | R | S | R | R | R | S | R | S | S | S | S | S | S | S | S | S | S | S |
| 3086 | C (pSg) | 4 | ESBL | + | - | R | R | R | S | R | R | R | S | R | R | S | S | S | S | S | S | S | S | S | S |
| 3087 | C (pSg) | 4 | ESBL | + | - | R | R | R | S | R | R | R | S | R | S | S | S | S | S | S | S | S | S | S | S |
| 3088 | C (pSg) | 4 | ESBL | + | - | R | R | R | S | R | R | R | S | R | S | S | S | S | S | S | S | S | S | S | S |
| 3089 | C (pSg) | 4 | ESBL | + | - | R | R | R | S | R | R | R | S | R | S | S | S | S | S | S | S | S | S | S | S |
| 3100 | C (pSg) | 5 | ESBL | + | + | R | R | R | R | R | R | R | S | R | S | S | S | S | S | S | R | S | S | S | R |
| 3101 | C (pSg) | 5 | ESBL | + | - | R | R | R | R | R | R | R | S | R | R | S | S | S | S | S | S | S | S | S | R |
| 3102 | C (pSg) | 5 | ESBL | + | - | R | R | R | S | R | R | R | S | R | S | S | S | S | S | S | S | S | S | S | R |
| 3103 | C (pSg) | 5 | ESBL | + | + | R | R | R | S | R | R | R | S | R | S | S | S | S | S | S | R | S | S | S | R |
| 3115 | C (pSg) | 6 | ESBL | + | + | R | R | R | R | R | R | R | S | R | S | S | S | S | S | S | R | S | S | S | R |
| 3116 | C (pSg) | 6 | ESBL | + | - | R | R | R | S | R | R | R | S | R | R | S | S | S | S | S | S | S | S | S | R |
| 3117 | C (pSg) | 6 | ESBL | + | - | R | R | R | S | R | R | R | S | S | R | S | S | S | S | R | S | S | S | S | R |
| 3118 | C (pSg) | 6 | ESBL | + | + | R | R | R | R | R | R | R | S | R | R | S | S | S | R | R | R | S | S | S | R |
| 2910 | D (pSg) | 1 | ESBL | + | - | R | R | R | S | R | R | R | S | R | S | S | S | S | S | S | S | S | S | S | R |
| 2911 | D (pSg) | 1 | ESBL | + | - | R | R | R | S | R | R | R | S | R | S | S | S | S | S | S | S | S | S | S | S |
| 2912 | D (pSg) | 1 | ESBL | + | - | R | R | R | S | R | R | R | S | R | S | S | S | S | S | S | S | S | S | S | R |
| 2913 | D (pSg) | 1 | ESBL | + | - | R | R | R | S | R | R | R | S | R | S | S | S | S | S | S | S | S | S | S | S |
| 2950 | D (pSg) | 2 | ESBL | + | - | R | R | R | S | R | R | R | S | R | S | S | S | S | S | S | S | S | S | S | R |
| 2951 | D (pSg) | 2 | ESBL | + | - | R | R | R | S | R | R | R | S | R | R | S | S | S | S | S | S | S | S | S | R |
| 2952 | D (pSg) | 2 | ESBL | + | - | R | R | R | S | R | R | R | S | R | S | S | S | S | S | S | S | S | S | S | R |
| 2953 | D (pSg) | 2 | ESBL | + | - | R | R | R | S | R | R | R | S | R | R | S | S | S | S | S | S | S | S | S | S |
| 2989 | D (pSg) | 3 | ESBL | + | - | R | R | R | S | R | R | R | S | R | S | S | S | S | S | S | S | S | S | S | S |
| 2990 | D (pSg) | 3 | ESBL | + | - | R | R | R | S | R | R | R | S | R | S | S | S | S | S | S | S | S | S | S | R |
| 2991 | D (pSg) | 3 | ESBL | + | - | R | R | R | S | R | R | R | S | R | S | S | S | S | S | S | R | S | S | S | R |
| 2992 | D (pSg) | 3 | ESBL | + | + | R | R | R | S | R | R | R | S | R | S | S | S | S | S | S | S | S | S | S | R |
| 3090 | D (pSg) | 4 | ESBL | + | - | R | R | R | S | R | R | R | S | R | S | S | S | S | S | S | S | S | S | S | R |
| 3091 | D (pSg) | 4 | ESBL | + | - | R | R | R | S | R | R | R | S | R | S | S | S | S | S | S | S | S | S | S | R |
| 3092 | D (pSg) | 4 | ESBL | + | + | R | R | R | S | R | R | R | S | R | S | S | S | S | S | S | R | S | S | S | R |
| 3093 | D (pSg) | 4 | ESBL | + | - | R | R | R | S | R | R | R | S | R | S | S | S | S | S | S | S | S | S | S | R |
| 3105 | D (pSg) | 5 | ESBL | + | - | R | R | R | S | R | R | R | S | R | R | S | S | S | S | S | S | S | S | S | S |
| 3106 | D (pSg) | 5 | ESBL | + | - | R | R | R | S | R | R | R | S | R | R | S | S | S | S | S | S | S | S | S | S |
| 3107 | D (pSg) | 5 | ESBL | + | - | R | R | R | S | R | R | R | S | R | S | S | S | S | S | S | S | S | S | S | R |
| 3108 | D (pSg) | 5 | ESBL | + | - | R | R | R | S | R | R | R | S | R | S | S | S | S | S | S | S | S | S | S | R |
| 3119 | D (pSg) | 6 | ESBL | + | - | R | R | R | S | R | R | R | S | R | S | S | S | S | S | S | S | S | S | S | R |
| 3120 | D (pSg) | 6 | ESBL | + | - | R | R | R | S | R | R | R | S | R | R | S | S | S | S | S | S | S | S | S | R |
| 3121 | D (pSg) | 6 | COL | + | + | R | R | R | S | R | R | R | S | R | R | S | S | S | S | S | R | S | S | R | R |
| 3123 | D (pSg) | 6 | ESBL | + | - | R | R | R | S | R | R | R | S | R | S | S | S | S | S | S | S | S | S | S | R |
| 3124 | D (pSg) | 6 | ESBL | + | - | R | R | R | S | R | R | R | S | R | S | S | S | S | S | S | S | S | S | S | R |
